# 

**Published:** 1872-02

**Authors:** 


EDITED BY

PROF. J. TAFT.

FEBRUARY, 1872.

MONTHLY:

THREE DOLLARS PER ANNUM, IN ADVANCE. SINGLE COPIES TWENTY-FIVE CENTS.

CINCINNATI:

SPENCER & MOORE, PUBLISHERS,

OHIO DENTAL DEPOT, ROOM No. 1 PIKE'S OPERA HOUSE.

Wrightson & Co., Printers, 167 Walnut St., Cin.

